# Acute Effects of Three Recovery Interventions on Post-Practice Vertical Jump Force-Time Metrics in Female Basketball Players

**DOI:** 10.3390/jfmk11010044

**Published:** 2026-01-21

**Authors:** Dimitrije Cabarkapa, Damjana V. Cabarkapa, Dora Nagy, Richard Repasi, Tamas Laczko, Laszlo Ratgeber

**Affiliations:** 1 D2 Lab, 21000 Novi Sad, Serbia; 2 Jayhawk Athletic Performance Laboratory—Wu Tsai Human Performance Alliance, Department of Health, Sport and Exercise Sciences, University of Kansas, Lawrence, KS 66045, USA; 3 Faculty of Physical Education and Management in Sport, Singidunum University, 11010 Belgrade, Serbia; 4 Faculty of Health Sciences, Doctoral School of Health Sciences, University of Pecs, 7602 Pecs, Hungary; 5 Faculty of Health Sciences, Institute of Physiotherapy and Sport Sciences, University of Pecs, 7602 Pecs, Hungary; 6 Center for Basketball Methodology and Education, 7621 Pecs, Hungary; 7 Department of Sport Games, Hungarian University of Sports Science, 1123 Budapest, Hungary

**Keywords:** cold water immersion, cryotherapy, compression, force, power, performance

## Abstract

**Objectives**: The purpose of the present study was to investigate the acute effects of cold-water immersion (CWI), cryotherapy (CRT), and intermittent pneumatic compression (IPC) on lower-body neuromuscular performance in female basketball players. **Methods**: Eighteen athletes volunteered to participate (body mass = 63.0 ± 7.2 kg; height = 171.4 ± 6.5 cm; age = 16.4 ± 1.2 years), completing testing at three time points: (i) pre-practice, (ii) post-practice, and (iii) 45–60 min following a randomly assigned recovery intervention. At each time point, athletes performed three countermovement vertical jumps on a dual force plate system sampling at 1000 Hz (VALD Performance). To standardize external load across groups, all players wore inertial measurement units (Kinexon). **Results**: The two-way repeated measures ANOVA showed no statistically significant interaction (*p* > 0.05) between the three testing time points and recovery modalities for any of the analyzed variables. However, a significant main effect of time was observed, with 13 of 20 force-time metrics (65%), including jump height, reactive strength index-modified, contraction time, and concentric peak and mean force, declining post-recovery compared with pre-practice values, regardless of the recovery intervention applied. External load measures (e.g., total distance, number of jumps) remained consistent across groups. **Conclusions**: Overall, these findings suggest that CWI, CRT, and IPC were no more effective than passive recovery (i.e., control group) in mitigating post-practice declines in lower-body force and power-producing capacities.

## 1. Introduction

Basketball is a highly dynamic team sport that places significant neuromuscular demands on the body [1,2,3,4]. Athletes are required to repeatedly perform high-intensity actions such as accelerations, decelerations, changes in direction, and explosive jumps [1,2,3,4,5]. These movements are physically fatiguing and, if not managed properly, can significantly impair performance capabilities. Therefore, adequate recovery is essential not only to minimize accumulated fatigue but also to restore neuromuscular function, reduce the risk of injury, and maintain consistent performance throughout training and competition [6,7].

Given the physical and physiological demands of competitive sports, a considerable number of recovery strategies have been used to manage fatigue and reduce the risk of developing injuries. Some of the most commonly implemented recovery methods in basketball include cryotherapy (CRT) [8,9,10,11], compression garments [12,13], intermittent pneumatic compression [14,15,16], massage [9,17], supplementation [18,19], hydration [20], sleep [21,22], and some forms of mindfulness techniques [5,23]. For example, sleep has long been recognized as one of the most important recovery strategies, with a substantial influence on athletes’ overall readiness and performance [22]. Specifically, extended sleep duration (i.e., >10 h per night) has been associated with faster sprint times as well as improved free-throw and three-point shooting accuracy [5,22]. Also, proper timing of food and fluid intake, along with the use of dietary supplements such as L-glutamine, branched-chain amino acids, vitamins, and minerals, plays a vital role in supporting performance and reducing the risk of injuries [18,19,20,24,25].

Another form of recovery frequently employed in athletic settings is cold water immersion (CWI), shown to effectively reduce stress responses in athletes [5,9,10,11]. Specifically, significantly lower heart rate, blood lactate levels, and ratings of perceived exertion were observed for athletes who underwent the CWI treatment when compared to the control group [10]. In addition to these effects, CWI proved more effective than massage, compression garments, and stretching, with substantial recovery benefits observed in basketball players 24–72 h post-exercise [9,11,26]. Alongside cryotherapy, intermittent pneumatic compression (IPC) has emerged as another widely adopted method for recovery and rehabilitation [14,16]. By applying periodic external compression, IPC enhances venous and arterial blood flow, which may facilitate the removal of metabolic waste products and help attenuate delayed onset muscle soreness following intense exercise [16,27]. While IPC provides moderate improvements in the perceived levels of fatigue and soreness, its impact on restoring muscle mechanical functions and promoting the clearance of the muscle damage and inflammatory markers still remains unclear [14,15,27,28]. This uncertainty is consistent with the broader body of literature, where evidence on the effectiveness of recovery strategies remains inconclusive, with improvements often appearing to reflect psychological rather than physiological responses. Thus, further research is warranted to better understand their impact on performance and the underlying mechanisms of action.

With the rapid growth of interest in neuromuscular fatigue monitoring and the advancement of the innovative force plate technology, countermovement vertical jump (CMJ) assessment has become one of the most frequently performed tests in basketball [29,30,31,32]. Previous studies have indicated that CMJ variables are capable of detecting performance changes in basketball players pre-post training session [29,30,33], as well as throughout a span of competitive season [32,34]. For example, Cabarkapa et al. [30] found that following a two-hour practice session, professional basketball players experienced a notable decrease in force-time metrics within the concentric phase of the jumping motion (e.g., impulse, velocity, force, power), indicating fatigue-related declines in performance. In addition, Philipp et al. [32] observed that CMJ metrics varied significantly across the competitive basketball season. Specifically, from pre-season to non-conference play, athletes showed shorter eccentric and concentric phase durations, along with increases in concentric mean and peak force, eccentric braking rate of force development, and eccentric mean deceleration force [32]. However, as the season progressed, these measures declined as accumulated fatigue set in [32]. Also, it should be noted that jump height did not change significantly across the season, highlighting the importance of a comprehensive analysis of CMJ in order to better capture neuromuscular fatigue [32,35]. While the aforementioned findings confirm that the CMJ assessment is a valuable tool for monitoring neuromuscular fatigue and performance fluctuations in basketball players, further research is needed to determine how different recovery modalities affect CMJ outcomes.

Therefore, the aim of the present study was to investigate the acute impact of three commonly used post-exercise recovery modalities (i.e., CWI, CRT, and IPC) on lower-body force-time characteristics (e.g., concentric mean force, eccentric peak power, countermovement depth, and jump height) within a cohort of female basketball players, a population that remains relatively understudied within the scientific literature. Additionally, the study sought to determine which recovery modality elicits the most favorable neuromuscular responses relative to passive recovery, thereby advancing current understanding and informing evidence-based recovery practices in applied sport settings. Based on previously published research, it was hypothesized that all three recovery interventions could elicit improvements in lower-body force–time metrics following an intense basketball training session, and that declines in neuromuscular performance would be evident both immediately post-practice and at a delayed post-recovery time point.

## 2. Materials and Methods

### 2.1. Participants

This study employed a randomized mixed-design experimental approach, with recovery modality as a between-subjects factor and time as a within-subjects factor. Eighteen female basketball players (body mass = 63.0 ± 7.2 kg; height = 171.4 ± 6.5 cm; age = 16.4 ± 1.2 years) volunteered to participate in the present investigation. The inclusion criteria involved athletes that were: (i) current members of the national basketball academy teams in Hungary, (ii) had more than three years of basketball playing experience, (iii) were cleared to participate in team training sessions and games by their respective sports medicine staff, and (iv) participated in structured team strength and conditioning and training sessions more than four times per week six weeks before the testing. The exclusion criteria involved any athletes with musculoskeletal injuries that would limit or impair their basketball or jumping performance. The testing procedures performed in this investigation were conducted in accordance with the Declaration of Helsinki and were approved by the University’s Institutional Review Board.

### 2.2. Neuromuscular Performance

The CMJ testing procedures were completed at three different testing time points: (i) immediately prior to the official team practice (12:00 h), (ii) immediately following completion of the team practice, and (iii) 60 min following the completion of the randomly assigned recovery session (i.e., CWI, CRT, IPC, and control—CTRL). The athletes stepped on a dual uniaxial force plate system sampling at 1000 Hz (ForceDecks Max, VALD Performance, Brisbane, Australia) and performed three CMJs with no arm swing (i.e., hands on the hip during the entire movement). The between-jump rest interval was set to 15 s, and the system was zeroed between each athlete, and the mean value across three jump trials was used for performance analysis purposes [1,2,30,36]. Throughout the testing procedures, the research assistants provided strong verbal encouragement. The athletes were instructed to quickly drop into a squat position at a self-selected depth and explosively push the ground to spring back up into a maximal-effort vertical jump. The force-time metrics examined in the present study were based on previously published research reports, as variables that demonstrated high practical applicability and solid levels of validity and reliability, within both eccentric and concentric phases of the CMJ [32,35,36,37]. Besides force and power metrics, the analysis included contraction time (i.e., time-to-takeoff), countermovement depth, jump height (i.e., impulse-momentum calculation), and reactive strength index (RSI)-modified (i.e., jump height/time to takeoff). The detailed definition of force-time metrics examined in the present investigation can be found in the VALD manual (https://support.vald.com/hc/en-au (accessed on 15 December 2025)).

### 2.3. Recovery Treatment

**Figure 1:**
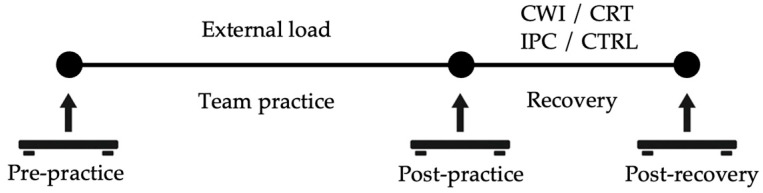
Graphical representation of the testing procedures.

The athletes were randomly assigned into one of four recovery groups: CWI (n = 5), CRT (n = 4), IPC (n = 5), and CTRL (i.e., passive rest or control condition; n = 4). The graphical representation of the testing timeline is presented in Figure 1. A simple random number generator was used to allocate participants into the different experimental groups. All recovery procedures were closely supervised by team medical staff members to ensure safety and protocol adherence. The recovery procedures were initiated approximately 20–30 min following the completion of the team training session. The CRT group underwent a whole-body cryotherapy session consisting of 90 s of exposure at −140 °C using a commercial cryochamber (Clasis Cryosauna, Nove Zamky, Slovakia). Before entry into the cryochamber, athletes were equipped with standard protective garments, including gloves, socks, and a headband to protect the extremities and avoid cold-related skin injuries. The CWI group performed a 10 min immersion in a water bath maintained at 14 °C (Chill Tubs Obsidian, Chesterfield, UK). Athletes were submerged to the level of the iliac crest (i.e., waist-deep) and remained seated and motionless throughout the session to standardize thermal exposure. Water temperature was continuously monitored using a calibrated thermometer to ensure consistent conditions. The athletes assigned to the IPC group completed a 15 min session using a commercial-grade pneumatic compression system (Normatec 3.0, Therabody Inc., Los Angeles, CA, USA). Compression sleeves were applied bilaterally, extending from the feet to the upper thighs. The system was operated at a high-intensity setting (i.e., Level 6 out of 7) and followed a standardized inflation-deflation cycle designed to promote venous blood return. On the other hand, the CTRL group received no active recovery treatment. Instead, athletes rested quietly in a supine position for 15 min in a climate-controlled room maintained at 21–23 °C. The athletes were instructed to remain still, avoid conversation, and refrain from using electronic devices to minimize cognitive/sensory stimulation. This passive recovery condition served as a baseline comparison for the active recovery interventions (i.e., CWI, CRT, and IPC).

### 2.4. External Load

During the training session, athletes wore an inertial measurement unit (Kinexon, Precision Technologies, Munich, Germany) to ensure comparable external training loads across all groups. This technology has been demonstrated as a valid testing modality for tracking walking, jogging, sprinting, jumping, as well as change in direction movements in team sports such as basketball [38,39]. The device operated at a sampling frequency of 20 Hz and was positioned between the scapulae using a tightly fitted vest or integrated into a sports bra to minimize movement artifacts. Data collection began 2–3 min before the start of the training session and ended immediately upon its completion. The external load variables analyzed in this study were selected based on previously published research reports [40,41], and included: high-speed running distance, distance per minute, accumulated acceleration load (AAL), average movement speed, and total counts of sprints and jumps. The official team training session lasted 60 min and followed a standardized non-contact format administered by the team’s coaching staff. The primary aim of the training session was focused on technical and tactical skill development, incorporating individual offensive drills (1-on-0), small-group activities (3-on-0), and full-team offensive and defensive sequences (5-on-0 and 5-on-5). To control for potential confounding variables, all testing procedures were performed under standardized conditions.

### 2.5. Statistical Analysis

Shapiro–Wilk’s test was used to confirm that the assumption of normality was not violated. Descriptive statistics, mean and standard deviation ($\bar{x}$ ± SD), were calculated for each dependent variable examined in the present investigation. A one-way analysis of variance (ANOVA) was used to examine between-group differences in external load metrics (i.e., CWI, CRT, IPC, and CTRL). A two-way repeated measures ANOVA was used to examine the presence of statistically significant main effects and interactions between treatment type and testing time point (i.e., pre-practice, post-practice, and post-recovery). When a significant main effect or interaction was observed, follow-up paired-sample *t*-tests were conducted using the LSD method. This method was purposely employed to identify the specific sources of difference, as it offers increased sensitivity for detecting subtle performance changes, which may be more appropriate for an applied sports setting. Statistical significance was set a priori to *p* < 0.05. All statistical analyses were completed with SPSS (Version 26.0; IBM Corp., Armonk, NY, USA).

## 3. Results

No statistically significant interaction effect between the testing time and treatment modality used was found in any force-time metric of interest, within both eccentric and concentric phases of the jumping motion: body weight (F_[6,28]_ = 0.635; *p* = 0.701), jump height (F_[6,28]_ = 0.508; *p* = 0.797), RSI-modified (F_[6,28]_ = 0.448; *p* = 0.840), contraction time (F_[6,28]_ = 0.857; *p* = 0.538), concentric peak power (F_[6,28]_ = 0.659; *p* = 0.683), concentric mean power (F_[6,28]_ = 0.296, *p* = 0.934), concentric peak velocity (F_[6,28]_ = 0.679; *p* = 0.668), concentric peak force (F_[6,28]_ = 0.905; 0.505), concentric mean force (F_[6,28]_ = 0.396; *p* = 0.876), concentric impulse (F_[6,28]_ = 0.640; *p* = 0.698), eccentric peak force (F_[6,28]_ = 0.678; *p* = 0.669), eccentric mean force (F_[6,28]_ = 0.586; *p* = 0.738), eccentric peak velocity (F_[6,28]_ = 1.247; *p* = 0.313), eccentric peak power (F_[6,28]_ = 1.937; *p* = 0.110), eccentric mean power (F_[6,28]_ = 1.643; *p* = 0.172), eccentric breaking impulse (F_[6,28]_ = 0.959; *p* = 0.512), braking phase duration (F_[6,28]_ = 0.358; *p* = 0.899), eccentric duration (F_[6,28]_ = 1.167; *p* = 0.353), concentric duration (F_[6,28]_ = 0.381; *p* = 0.885), and countermovement depth (F_[6,28]_ = 0.636; *p* = 0.700).

On the other hand, a statistically significant effect of time has been observed in jump height F_[2,28]_ = 18.424; *p* < 0.001), RSI-modified (F_[2,28]_ = 12.937; *p* < 0.001), contraction time (F_[2,28]_ = 4.717; *p* = 0.017), concentric peak power (F_[2,28]_ = 10.838; *p* < 0.001), concentric mean power (F_[2,28]_ = 9.224; *p* < 0.001), concentric peak velocity (F_[2,28]_ = 15.730; *p* = 0.002), concentric peak force (F_[2,28]_ = 3.751; *p* = 0.035), concentric mean force (F_[2,28]_ = 6.126; *p* = 0.006), concentric impulse (F_[2,28]_ = 16.567; *p* < 0.001), eccentric peak force (F_[2,28]_ = 6.984; *p* = 0.003), eccentric peak velocity (F_[2,28]_ = 4.668; *p* = 0.017), eccentric peak power (F_[2,28]_ = 5.645; *p* = 0.009), eccentric mean power (F_[2,28]_ = 4.081; *p* = 0.028), eccentric braking impulse (F_[2,28]_ = 5.290; *p* = 0.011), braking phase duration (F_[2,28]_ = 5.965; *p* = 0.007), and eccentric duration (F_[2,28]_ = 5.680; *p* = 0.008). Yet, no within-subject differences across three testing time points were detected in body mass (F_[2,28]_ = 0.493; *p* = 0.616), eccentric mean force (F_[2,28]_ = 1.775; *p* = 0.188), concentric duration (F_[2,28]_ = 1.160; *p* = 0.208), and countermovement depth (F_[2,28]_ = 0.037; *p* = 0.963).

Specifically, a notable decline in the following force-time metrics was observed post-recovery when compared to pre-practice and post-practice values in jump height (*p* < 0.001; *p* < 0.001), RSI-modified (*p* = 0.002; *p* = 0.003), contraction time (*p* = 0.020; *p* = 0.040), concentric peak power (*p* = 0.003; *p* = 0.002), concentric mean power (*p* = 0.007; *p* = 0.002), concentric peak velocity (*p* < 0.001; *p* < 0.001), concentric mean force (*p* = 0.019; *p* = 0.016), concentric impulse (*p* < 0.001; *p* < 0.001), eccentric peak force (*p* = 0.016; *p* = 0.008), eccentric peak velocity (*p* = 0.039; *p* = 0.036), eccentric peak power (*p* = 0.018; *p* = 0.011), eccentric braking impulse (*p* = 0.011; *p* = 0.041), and braking phase duration (*p* = 0.016; *p* = 0.013), respectively. Additionally, statistically significant differences were observed in concentric peak force (*p* = 0.045), eccentric mean power (*p* = 0.027), and eccentric duration (*p* = 0.010) between pre-practice and post-recovery testing timepoints. The detailed results for each force-time metric are presented in Table 1, Table 2 and Table 3.

In addition, no statistically significant between-group differences have been found across found different recovery strategies (i.e., CWI, CRT, IPC, and CTRL) in high speed distance (F_[3,14]_ = 0.512; *p* = 0.681), distance per minute (F_[3,14]_ = 0.049; *p* = 0.985), AAL (F_[3,14]_ = 0.141; *p* = 0.934), average running speed (F_[3,14]_ = 0.065; *p* = 0.977), total number of sprints (F_[3,14]_ = 0.370; *p* = 0.776), and total number of jumps (F_[3,14]_ = 1.721; *p* = 0.209). The detailed results, including means and standard deviations, for each external load metric can be found in Table 4.

## 4. Discussion

The aim of the present study was to examine the acute effects of three commonly used post-exercise recovery modalities (i.e., CWI, CRT, and IPC) on lower-body neuromuscular performance characteristics in female basketball players. To the best of our knowledge, this is the first study to embed a detailed analysis of force-time metrics while simultaneously controlling for external load differences, conducted specifically on female basketball players, an understudied population in the scientific literature. Contrary to our initial hypothesis, no statistically significant interaction effects were observed between testing time point (i.e., pre-practice, post-practice, and post-recovery) and recovery modality (i.e., CWI, CRT, and IPC) for any variables of interest. However, the main effect of time was significant. Specifically, thirteen out of twenty force-time metrics (65%), including jump height, RSI-modified, contraction time, and concentric peak and mean force, showed a notable decline in magnitude post-recovery compared with both pre-practice and post-practice values, regardless of the recovery modality applied. Moreover, external load (e.g., total distance, number of jumps) was identical across athletes in all three recovery groups, and no differences in body mass were observed, reducing the likelihood that these factors influenced the reported force-time outcomes.

A considerable amount of scientific literature has examined the impact of CWI on athlete recovery across a variety of performance tests [9,11,42,43]. For example, when examining a mixed cohort of male and female basketball players, Delextrat et al. [9] reported that CWI improved jump performance but had no effect on repeated-sprint ability, with jump performance remaining greater at both baseline and after CWI compared with the CTRL condition. Conversely, Montgomery et al. [11] observed a greater decrease in vertical jump height across a three-day basketball tournament in the CWI group (7.2%; 67.2 ± 8.4 vs. 61.6 ± 6.5 cm) compared with the CTRL group (2.6%; 61.1 ± 8.2 vs. 58.7 ± 6.7 cm). While both of these studies demonstrated that exercise induced declines in jump performance, consistent with the findings of the present investigation, a key difference emerged. In the present study, the reduction in jump height, along with decreases in force and power-producing capacities within both eccentric and concentric phases of the CMJ, occurred not immediately post-practice but post-recovery, with no differences observed between the CWI and CTRL conditions. A possible explanation for these discrepancies lies in the type and intensity of the exercise stimulus (e.g., three-day tournament vs. single practice session) as well as the duration and intensity of CWI exposure [44]. Supporting this, De Nardi et al. [45] investigated a group of youth soccer players across a four-day training period and found no significant differences between CWI (i.e., 8 min at 15 °C) and CTRL condition (i.e., passive recovery) in repeated-sprint ability, shuttle run, small-sided games, or creatine kinase (i.e., marker of muscle damage), which increased in both groups. As highlighted by Tipton et al. [44], the duration and intensity of exposure may play a critical role in determining CWI effectiveness, potentially offering a further explanation for the divergent results across studies. For instance, Montgomery et al. [11] used an intermittent protocol consisting of five one-minute immersions at 11 °C, whereas in this study athletes underwent a single continuous ten-minute immersion at 14 °C. Another factor often considered is age, as thermoregulatory responses to CWI may differ between younger and older athletes (23.0 ± 3.0 vs. 16.4 ± 1.2 years). However, while further research is warranted on this topic, Glickman et al. [46] reported no significant differences in thermosensitivity between young and older individuals when exposed to CWI.

Similarly to CWI, previous research has reported mixed results regarding the effectiveness of CRT in enhancing post-exercise recovery [47,48,49,50]. For instance, Russell et al. [49] reported that a single CRT session (i.e., 30 s at −60 °C and 120 s at −135 °C) following a repeated-sprint exercise protocol (i.e., 15 × 30 m sprints with 10 m forced deceleration) increased post-exercise testosterone concentrations compared with a CTRL condition, yet no differences were observed in CMJ performance (e.g., concentric power) or blood lactate levels. Similarly, Vieira et al. [50] found that whole-body CRT (i.e., 3 min at −110 °C) had no effect on vertical jump performance (i.e., jump height, concentric power, and velocity) after a high-intensity interval protocol, with both CRT and CTRL groups showing notable pre-post-performance declines (i.e., 2.8–10.1%). These findings align with the present investigation, where no differences were observed between a 90 s CRT exposure at −140 °C and the CTRL group (i.e., passive recovery). However, other studies have demonstrated positive effects of repeated whole-body CRT exposures, with multiple sessions more consistently associated with improvements in recovery from pain and reductions in markers of inflammation and muscle damage such as TNF-α, IL-2, IL-6, and IL-8 [51]. A possible explanation for these discrepancies is that the athletes in the present study were not consistently exposed to CRT, which may have reduced the effectiveness of a single application and contributed to the lack of improvements in lower-body neuromuscular performance characteristics such as CMJ eccentric and concentric force and power outputs. Furthermore, as noted previously with CWI, the wide variability in protocols, exposure timing relative to exercise, and differences in temperature, duration, and frequency of application make firm recommendations for CRT use premature at present [47,48,51].

In line with the overall findings of the present study, the reduction in both concentric and eccentric variables relative to pre-practice values was similar between the IPC and CTRL (i.e., passive recovery) conditions. These results suggest that IPC does not provide a superior means of optimizing neuromuscular recovery, which is consistent with previously published scientific literature [5,15]. While some studies have reported positive effects of IPC on perceptual measures (e.g., soreness, perceived recovery) and biochemical markers (e.g., reductions in inflammatory cytokines), performance outcomes remain inconsistent [28,52,53,54]. Evidence further suggests that protocols lasting 20–30 min at pressures around 80 mmHg may be most commonly used to optimize recovery [15], yet even under these conditions, neuromuscular benefits appear limited. For example, Khan et al. [55] reported that IPC lowered heart rate during the first minute of recovery after submaximal exercise (i.e., treadmill running) in collegiate basketball players, while Collins et al. [56] found potentially beneficial effects on biomarkers (i.e., increase in immunoglobulin-A and attenuated decline in testosterone-to-cortisol ratio) of recovery in rugby, soccer, and basketball athletes without corresponding improvements in jump height or other neuromuscular outcomes. Specifically, CMJ jump height, with an arm swing, experiences trivial changes pre-post recovery in both IPC group (40.9 ± 7.8 vs. 40.4 ± 7.6 cm) and CTRL group (34.1 ± 4.1 vs. 33.8 ± 5.3 cm). While no differences were observed between IPC and CTRL treatment modalities, which is consistent with previous research, the present study differed in that both groups demonstrated a decline in jump height post-recovery compared with pre-practice values. In addition, although IPC may provide short-term relief from delayed-onset muscle soreness, it does not seem to attenuate strength loss or prevent exercise-induced muscle damage [57]. Taken together, these findings suggest that IPC may support cardiovascular and perceptual recovery but does not preserve explosive force or power-producing capabilities in the acute post-exercise period.

An interesting observation that emerges from the scientific literature is that, although findings on objective post-recovery performance outcomes are mixed, there is consistent evidence of improvements in subjective perceptions of recovery [5,15,42,47]. For example, Delextrat et al. [9] reported that CWI led to notable reductions in perceived fatigue in a mixed cohort of male and female basketball players, while Leeder et al. [42] found that CWI reduced perceptions of muscle soreness by an average of 16%. Similarly, Rossato et al. [58] showed that cryotherapy applied after eccentric downhill running significantly reduced delayed-onset muscle soreness and helped reestablish homeostasis in markers of muscle damage. In line with this, Maia et al. [15] indicated that lower-limb IPC may also reduce perceived fatigue and soreness levels, suggesting potential recovery benefits. However, these perceptual measures were not examined in the present study, which represents a limitation. Thus, we cannot determine whether CWI, CRT, or IPC has a superior impact on subjective recovery compared with CTRL (i.e., passive recovery). Nevertheless, when considering previously published research collectively, recovery modalities appear to offer reliable improvements in perceived recovery, though whether these benefits outweigh the logistical challenges of implementation, particularly with CWI, remains a decision for applied sports practitioners and warrants further research.

Last but not least, it is important to mention that no differences pre-post practice were observed in lower-body neuromuscular performance characteristics in both outcome metrics (e.g., jump height, RSI-modified) as well as strategy metrics (i.e., contraction time, eccentric and concentric duration, and countermovement depth). This is consistent with the previously published research reports that examined acute fatigue-induced changes in athlete performance [1,2,30,36]. For instance, when studying a cohort of professional basketball players, no differences in eccentric and concentric peak and mean force and power were observed pre-post an official basketball game [1]. In a follow-up investigation, the same group of authors found that the same force-time metrics tend to remain consistent even during a game, from the first quarter to the last [36]. However, it should be noted that fatigue is a complex and multifaceted phenomenon. While it may not always be visible in traditional outcome or strategy metrics, it can still manifest in more subtle neuromuscular, perceptual, or biochemical domains that were not captured in the present study [59]. In addition, although no immediate pre-post-practice changes were observed in lower-body neuromuscular performance, thirteen out of twenty force-time metrics (65%) showed a considerable decline post-recovery compared with pre-practice values, regardless of the recovery modality applied (i.e., CWI, CRT, IPC). Supporting this pattern, Yoshida et al. [60] reported the greatest reductions in CMJ relative peak and mean force, time-to-peak force, and average rate of force development in well-trained collegiate basketball players approximately two hours post-exercise. In the present study, CMJ testing was conducted 45–60 min following practice, which may explain why decrements were detected post-recovery rather than immediately after training. Thus, the decline in the aforementioned CMJ force-time metrics could be attributed to a delayed fatigue response to the exercise stimulus, in this case, an intense basketball practice session. However, this assumption warrants further research to clarify the time course of neuromuscular fatigue in basketball players and to examine how recovery strategies, such as those investigated in the present study, may influence delayed performance decrements.

While providing a deeper insight into the impact of some of the most commonly used recovery strategies in sports (i.e., CWI, CRT, IPC), this study is not without limitations. The relatively small sample size and focus on a single cohort of female basketball players may limit the generalizability of the findings to other populations, including male athletes or players from different competitive levels (e.g., collegiate, professional). Additionally, only acute effects were examined, and recovery was assessed within a short time window (i.e., 60 min post-practice), which may not fully capture the longer-term dynamics of neuromuscular fatigue or recovery responses. Also, crossover design was not implemented due to practical and methodological constraints in an applied team-sport setting. In addition, perceptual and biochemical markers, including menstrual cycle status, were not measured, preventing a more comprehensive understanding of the multidimensional nature of fatigue and recovery. Therefore, future research should investigate larger and more diverse athlete populations, employ longitudinal designs to track recovery over extended time periods, and integrate both objective and subjective measures of fatigue. Moreover, examining the interaction between different recovery strategies and their potential cumulative effects across a full competitive season span would provide valuable applied insights for sports practitioners.

## 5. Conclusions

The findings of the present study revealed no statistically significant interaction effects between recovery modality (i.e., CWI, CRT, IPC) and testing time points, with all groups demonstrating similar responses when compared to the CTRL condition (i.e., passive recovery). Instead, a main effect of time was identified, as thirteen out of twenty force-time metrics (65%) declined post-recovery compared with pre-practice values (e.g., jump height, RSI-modified, contraction time, eccentric peak force and velocity, and concentric mean and peak power). These results indicate that the applied recovery modalities did not prevent decrements in force and power-producing capacities, and the lack of differences between treatments and the CTRL condition may be attributed to the reliance on objective performance measures. Importantly, although objective neuromuscular outcomes showed no benefits, previous research has reported improvements in subjective recovery markers (e.g., perceived fatigue and muscle soreness), which were not assessed in the present study. This suggests that acute recovery effects may be primarily perceptual rather than reflected in force-time characteristics.

## Figures and Tables

**Table 1:** Descriptive statistics for the force-time metric within the concentric phase of the CMJ.

Variable (Unit)	Pre-Practice	Post-Practice	Post-Recovery
CON mean force (N)			
CTRL	1238.5 ± 227.3	1223.5 ± 205.1	1181.8 ± 132.5 *
CWI	1124.2 ± 142.9	1120.2 ± 139.9	1091.0 ± 150.9 *
CRT	1143.8 ± 138.7	1157.3 ± 159.6	1127.0 ± 185.2 *
IPC	1177.2 ± 105.2	1152.2 ± 92.9	1133.0 ± 94.6 *
CON peak force (N)			
CTRL	1557.3 ± 309.9	1518.0 ± 283.1	1446.5 ± 130.9 ^#^
CWI	1361.0 ± 183.5	1362.4 ± 175.6	1335.0 ± 177.2 ^#^
CRT	1365.0 ± 151.5	1373.8 ± 182.2	1371.3 ± 204.7 ^#^
IPC	1543.2 ± 208.5	1518.4 ± 185.6	1481.0 ± 195.5 ^#^
CON mean power (W)			
CTRL	1593.0 ± 483.5	1550.8 ± 433.2	1465.3 ± 296.0 *
CWI	1379.2 ± 136.9	1384.0 ± 113.4	1306.6 ± 137.1 *
CRT	1463.3 ± 140.8	1474.0 ± 209.2	1395.5 ± 231.8 *
IPC	1527.0 ± 114.9	1480.8 ± 116.7	1402.8 ± 117.8 *
CON peak power (W)			
CTRL	2863.8 ± 663.7	2804.9 ± 628.7	2688.9 ± 460.6 *
CWI	2577.6 ± 273.2	2572.1 ± 298.8	2421.2 ± 369.0 *
CRT	2661.3 ± 215.9	2692.2 ± 295.2	2626.8 ± 321.4 *
IPC	2739.5 ± 254.0	2689.1 ± 316.2	2534.1 ± 284.6 *
CON peak velocity (m/s)			
CTRL	2.40 ± 0.25	2.37 ± 0.24	2.33 ± 0.19 *
CWI	2.35 ± 0.16	2.35 ± 0.16	2.28 ± 0.13 *
CRT	2.43 ± 0.14	2.42 ± 0.12	2.38 ± 0.08 *
IPC	2.43 ± 0.21	2.40 ± 0.23	2.31 ± 0.16 *
CON impulse (Ns)			
CTRL	146.5 ± 25.4	143.6 ± 24.3	141.3 ± 22.9 *
CWI	138.8 ± 18.7	137.4 ± 19.4	132.8 ± 21.7 *
CRT	141.9 ± 9.2	141.7 ± 13.9	138.9 ± 14.1 *
IPC	141.8 ± 15.8	138.4 ± 18.9	133.4 ± 18.5 *
CON duration (s)			
CTRL	0.249 ± 0.042	0.248 ± 0.037	0.257 ± 0.031
CWI	0.277 ± 0.044	0.274 ± 0.040	0.280 ± 0.041
CRT	0.268 ± 0.024	0.261 ± 0.028	0.273 ± 0.030
IPC	0.254 ± 0.058	0.258 ± 0.055	0.256 ± 0.054

Note: CON—concentric; CMJ—countermovement vertical jump; (*)—significantly different when compared to pre-practice and post-practice testing timepoints (*p* < 0.05); (#)—significantly different when compared to pre-practice testing timepoint (*p* < 0.05); CTRL—control; CRT—cryotherapy; CWI—cold water immersion; IPC—intermittent pneumatic compression.

**Table 2:** Descriptive statistics for the force-time metric within the eccentric phase of the CMJ.

Variable (Unit)	Pre-Practice	Post-Practice	Post-Recovery
ECC mean force (N)			
CTRL	631.8 ± 62.4	630.8 ± 60.5	631.0 ± 61.7
CWI	618.6 ± 118.5	618.5 ± 118.3	617.4 ± 118.4
CRT	610.3 ± 72.9	610.5 ± 73.6	609.5 ± 74.1
IPC	605.2 ± 62.9	606.0 ± 62.8	605.2 ± 63.1
ECC peak force (N)			
CTRL	1493.8 ± 315.3	1447.8 ± 270.4	1349.8 ± 145.6 *
CWI	1297.2 ± 137.1	1305.4 ± 135.5	1281.0 ± 159.9 *
CRT	1364.0 ± 154.6	1334.5 ± 219.0	1244.9 ± 288.7 *
IPC	1530.8 ± 207.8	1489.2 ± 190.8	1445.6 ± 210.0 *
ECC mean power (W)			
CTRL	402.5 ± 80.4	389.5 ± 79.9	354.5 ± 87.4 ^#^
CWI	400.6 ± 80.4	395.0 ± 64.4	399.0 ± 98.7 ^#^
CRT	389.2 ± 42.8	372.0 ± 62.6	341.0 ± 84.2 ^#^
IPC	402.6 ± 69.3	401.2 ± 74.9	406.4 ± 71.8 ^#^
ECC peak power (W)			
CTRL	1246.8 ± 321.4	1254.5 ± 380.2	1011.3 ± 274.5 *
CWI	1105.2 ± 230.7	1116.4 ± 136.8	1078.2 ± 251.4 *
CRT	1064.5 ± 77.3	1042.3 ± 239.3	891.3 ± 292.6 *
IPC	1294.4 ± 285.8	1204.6 ± 332.2	1266.0 ± 304.9 *
ECC peak velocity (m/s)			
CTRL	1.28 ± 0.13	1.26 ± 0.15	1.14 ± 0.18 *
CWI	1.28 ± 0.10	1.28 ± 0.11	1.26 ± 0.14 *
CRT	1.24 ± 0.09	1.21 ± 0.15	1.09 ± 0.31 *
IPC	1.35 ± 0.19	1.32 ± 0.22	1.34 ± 0.18 *
ECC impulse (Ns)			
CTRL	45.3 ± 9.3	44.4 ± 9.4	41.9 ± 10.0 *
CWI	46.7 ± 10.7	44.9 ± 6.1	42.9 ± 10.3 *
CRT	42.2 ± 1.6	41.3 ± 5.2	35.8 ± 11.0 *
IPC	46.2 ± 9.8	45.9 ± 7.9	46.1 ± 8.2 *
ECC duration (s)			
CTRL	0.396 ± 0.038	0.413 ± 0.033	0.453 ± 0.029 ^#^
CWI	0.460 ± 0.083	0.460 ± 0.063	0.464 ± 0.051 ^#^
CRT	0.445 ± 0.020	0.458 ± 0.024	0.469 ± 0.013 ^#^
IPC	0.413 ± 0.066	0.424 ± 0.057	0.431 ± 0.066 ^#^

Note: ECC—eccentric; CMJ—countermovement vertical jump; (*)—significantly different when compared to pre-practice and post-practice testing timepoints (*p* < 0.05); (#)—significantly different when compared to pre-practice testing timepoint (*p* < 0.05); CTRL—control; CRT—cryotherapy; CWI—cold water immersion; IPC—intermittent pneumatic compression.

**Table 3:** Descriptive statistics for CMJ outcome and strategy metrics, including athletes’ body mass.

Variable (Unit)	Pre-Practice	Post-Practice	Post-Recovery
Body mass (kg)			
CTRL	64.2 ± 6.3	64.2 ± 6.2	64.3 ± 6.3
CWI	63.0 ± 12.1	63.0 ± 12.1	62.9 ± 12.1
CRT	62.1 ± 7.4	62.1 ± 7.5	62.1 ± 7.6
IPC	61.6 ± 6.5	61.7 ± 6.4	61.6 ± 6.4
Jump height (cm)			
CTRL	26.6 ± 6.0	25.6 ± 5.7	24.6 ± 4.5 *
CWI	25.3 ± 4.0	24.8 ± 4.1	23.2 ± 3.3 *
CRT	27.0 ± 3.4	26.7 ± 3.3	25.6 ± 2.2 *
IPC	27.1 ± 5.5	26.1 ± 5.7	24.2 ± 5.4 *
RSI-modified (ratio)			
CTRL	0.43 ± 0.14	0.39 ± 0.11	0.34 ± 0.06 *
CWI	0.36 ± 0.09	0.34 ± 0.09	0.31 ± 0.05 *
CRT	0.38 ± 0.04	0.37 ± 0.05	0.34 ± 0.03 *
IPC	0.41 ± 0.07	0.39 ± 0.06	0.35 ± 0.05 *
Contraction time (s)			
CTRL	0.645 ± 0.077	0.661 ± 0.059	0.710 ± 0.033 *
CWI	0.737 ± 0.127	0.734 ± 0.099	0.745 ± 0.089 *
CRT	0.714 ± 0.036	0.720 ± 0.051	0.742 ± 0.034 *
IPC	0.667 ± 0.123	0.682 ± 0.111	0.687 ± 0.117 *
Braking phase duration (s)			
CTRL	0.247 ± 0.041	0.250 ± 0.036	0.262 ± 0.015 *
CWI	0.287 ± 0.051	0.283 ± 0.041	0.295 ± 0.036 *
CRT	0.278 ± 0.010	0.281 ± 0.011	0.303 ± 0.022 *
IPC	0.247 ± 0.033	0.255 ± 0.033	0.261 ± 0.033 *
CMJ depth (cm)			
CTRL	25.2 ± 2.7	25.3 ± 2.0	25.1 ± 3.2
CWI	30.0 ± 6.2	29.6 ± 4.7	30.1 ± 5.8
CRT	28.3 ± 2.1	28.0 ± 2.9	26.9 ± 6.5
IPC	27.9 ± 7.6	28.5 ± 7.5	29.0 ± 8.0

Note: (*)—significantly different when compared to pre-practice and post-practice testing timepoints (*p* < 0.05); (CMJ—countermovement vertical jump; RSI—reactive strength index; CTRL—control; CRT—cryotherapy; CWI—cold water immersion; IPC—intermittent pneumatic compression.

**Table 4:** Between-group differences in the external load metric across four recovery strategies.

Variable (Unit)	CTRL	CWI	CRT	IPC
HS distance (m)	924.0 ± 99.8	943.2 ± 93.1	988.3 ± 98.3	925.8 ± 93.5
Distance (m/min)	89.0 ± 9.6	89.2 ± 13.5	90.1 ± 7.2	90.0 ± 13.2
AAL (au)	6.54 ± 0.59	6.82 ± 0.89	6.50 ± 0.58	6.6 ± 1.0
Speed (km/h)	5.26 ± 0.58	5.34 ± 0.83	5.46 ± 0.43	5.41 ± 0.79
Sprints (total)	55.8 ± 3.4	56.1 ± 8.2	54.5 ± 7.2	58.6 ± 4.4
Jumps (total)	22.7 ± 3.8	20.6 ± 6.1	21.3 ± 3.7	26.2 ± 6.9

Note: CTRL—control; CRT—cryotherapy; CWI—cold water immersion; IPC—intermittent pneumatic; HS—high speed; AAL—accumulated acceleration load; au—arbitrary unit.

## Data Availability

The data presented in this study are not publicly available due to privacy and ethical restrictions.
